# Informing the Future of Integrated Digital and Clinical Mental Health Care: Synthesis of the Outcomes From Project Synergy

**DOI:** 10.2196/33060

**Published:** 2022-03-09

**Authors:** Haley M LaMonica, Frank Iorfino, Grace Yeeun Lee, Sarah Piper, Jo-An Occhipinti, Tracey A Davenport, Shane Cross, Alyssa Milton, Laura Ospina-Pinillos, Lisa Whittle, Shelley C Rowe, Mitchell Dowling, Elizabeth Stewart, Antonia Ottavio, Samuel Hockey, Vanessa Wan Sze Cheng, Jane Burns, Elizabeth M Scott, Ian B Hickie

**Affiliations:** 1 Brain and Mind Centre The University of Sydney Camperdown Australia; 2 InnoWell Pty Ltd Sydney Australia

**Keywords:** mental health, technology, co-design, participatory research, health care reform, stakeholder participation, mobile phone

## Abstract

**Background:**

Globally, there are fundamental shortcomings in mental health care systems, including restricted access, siloed services, interventions that are poorly matched to service users’ needs, underuse of personal outcome monitoring to track progress, exclusion of family and carers, and suboptimal experiences of care. Health information technologies (HITs) hold great potential to improve these aspects that underpin the enhanced quality of mental health care.

**Objective:**

Project Synergy aimed to co-design, implement, and evaluate novel HITs, as exemplified by the InnoWell Platform, to work with standard health care organizations. The goals were to deliver improved outcomes for specific populations under focus and support organizations to enact significant system-level reforms.

**Methods:**

Participating health care organizations included the following: Open Arms–Veterans & Families Counselling (in Sydney and Lismore, New South Wales [NSW]); NSW North Coast headspace centers for youth (Port Macquarie, Coffs Harbour, Grafton, Lismore, and Tweed Heads); the Butterfly Foundation’s National Helpline for eating disorders; Kildare Road Medical Centre for enhanced primary care; and Connect to Wellbeing North Coast NSW (administered by Neami National), for population-based intake and assessment. Service users, families and carers, health professionals, and administrators of services across Australia were actively engaged in the configuration of the InnoWell Platform to meet service needs, identify barriers to and facilitators of quality mental health care, and highlight potentially the best points in the service pathway to integrate the InnoWell Platform. The locally configured InnoWell Platform was then implemented within the respective services. A mixed methods approach, including surveys, semistructured interviews, and workshops, was used to evaluate the impact of the InnoWell Platform. A participatory systems modeling approach involving co-design with local stakeholders was also undertaken to simulate the likely impact of the platform in combination with other services being considered for implementation within the North Coast Primary Health Network to explore resulting impacts on mental health outcomes, including suicide prevention.

**Results:**

Despite overwhelming support for integrating digital health solutions into mental health service settings and promising impacts of the platform simulated under idealized implementation conditions, our results emphasized that successful implementation is dependent on health professional and service readiness for change, leadership at the local service level, the appropriateness and responsiveness of the technology for the target end users, and, critically, funding models being available to support implementation. The key places of interoperability of digital solutions and a willingness to use technology to coordinate health care system use were also highlighted.

**Conclusions:**

Although the COVID-19 pandemic has resulted in the widespread acceptance of very basic digital health solutions, Project Synergy highlights the critical need to support equity of access to HITs, provide funding for digital infrastructure and digital mental health care, and actively promote the use of technology-enabled, coordinated systems of care.

## Introduction

### Mental Health Service Reform

In 2014, the National Mental Health Commission invited the Young and Well Cooperative Research Centre (CRC) to provide advice about digital mental health solutions for young people [1]. Following interviews with mental health and industry leaders, the Young and Well CRC promoted the view that there was an urgent need to broaden research and development (R&D) focus from standalone mental health apps, digital health records, and other simple technologies (Digital Health 1.0) to highly coordinated integration of health information technologies (HITs) within more effective health care delivery systems (HIT Digital Health 2.0). That is, new systems of care would integrate both conventional and novel in-clinic and web-based mental health services, thereby broadening the rapid provision of scalable forms of high-quality care.

As a result, the National Mental Health Commission recommended that HITs be considered a cornerstone of mental health service reform in Australia. This would require that the mental health system be overhauled to embed digital health within conventional in-clinic services [2]. More recently, this recommendation was echoed by the Australian Productivity Commission review of mental health. It calls for increased use of technology through a national digital platform, emphasizing initial assessment and referral processes, as well as increasing service user access to a broader range of interventions and supports [3]. However, there is still a considerable need for increased practical knowledge as to how to best incorporate HITs into clinical services, as well as how to capitalize on the aggregate information available from HITs that could be used to drive more effective coordination of care, enhanced allocation of clinical resources, faster identification of risk, and greater emphasis on achieving a higher proportion of improved health, social, and economic outcomes.

Over the past 20 years, our research group has actively advocated for the use of technology to drive enhanced mental health care, and broader system reform, in Australia [4-8]. Although there is a growing evidence base supporting the use of digital technologies, the integration of digital solutions in mental health services remains relatively rare. As an example, Titov et al [9] have reported success in integrating internet-delivered cognitive behavioral therapy as part of clinical care with a mental health professional in 5 clinics internationally. Unfortunately, far more studies highlight barriers to successful implementations of digital health solutions [10-13], and further research is required to identify the most effective ways in which to design and implement evidence-based HITs as part of clinical care at scale.

### Project Synergy

The Australian Government Department of Health commissioned Project Synergy in 2014 to be conducted by the Young and Well CRC in partnership with the University of Sydney’s Brain and Mind Centre. However, following the completion of the Young and Well CRC’s work in 2016, InnoWell, a joint venture enterprise between the University of Sydney and PwC (Australia), was established to deliver the remainder of the work. The overarching objective of Project Synergy is to explore how best to use such new HITs to drive mental health service reform toward delivering better outcomes for people experiencing mental ill-health [4].

Because of its scale, Project Synergy had 2 phases. Phase I (2014-2016; Aus $5.5 million [US $3.97 million]) investigated the use of digital (including smartphone) technologies to transform the provision of mental health services for Australian young people through co-designing, building, implementing, and evaluating prototypes with representative end user populations. These included young people along with their supportive others (eg, family, carers, and friends), health professionals, and service providers [4]. Four research studies were conducted on prototypical software and concepts that included the following: (1) a university-based health and well-being prototype [14]; (2) a synergized web-based system including a triage e-tool, health and well-being e-tool, and a *Mental Health eClinic* with a multidimensional web-based assessment, shared mental health dashboard, and *video visit* capabilities [15]; (3) wireframes for a tiered suicide risk management protocol [16]; (4) functionality for trusted supportive others [16-18]; (5) and a proof of concept study for implementing technologies into primary youth mental health services [19]. The details of these preliminary studies have been published previously [4].

The remainder of this paper focuses on phase II (2017-2021; Aus $30 million [US $21.63 million]), which concluded in June 2021. In phase II, prototypes from phase I were iterated into a beta version of a single web-based platform named the InnoWell Platform and implemented into both conventional in-clinic and web-based primary mental health services. The fundamental aim was articulated as delivering the right level of care to individuals upon their first presentation to service (*Right care, First time*) as underpinned by clinical stage, level of clinical need, and personal choice, with the aim of preventing progression to more serious complex and severe forms of illness.

### InnoWell Platform

**Figure 1:**
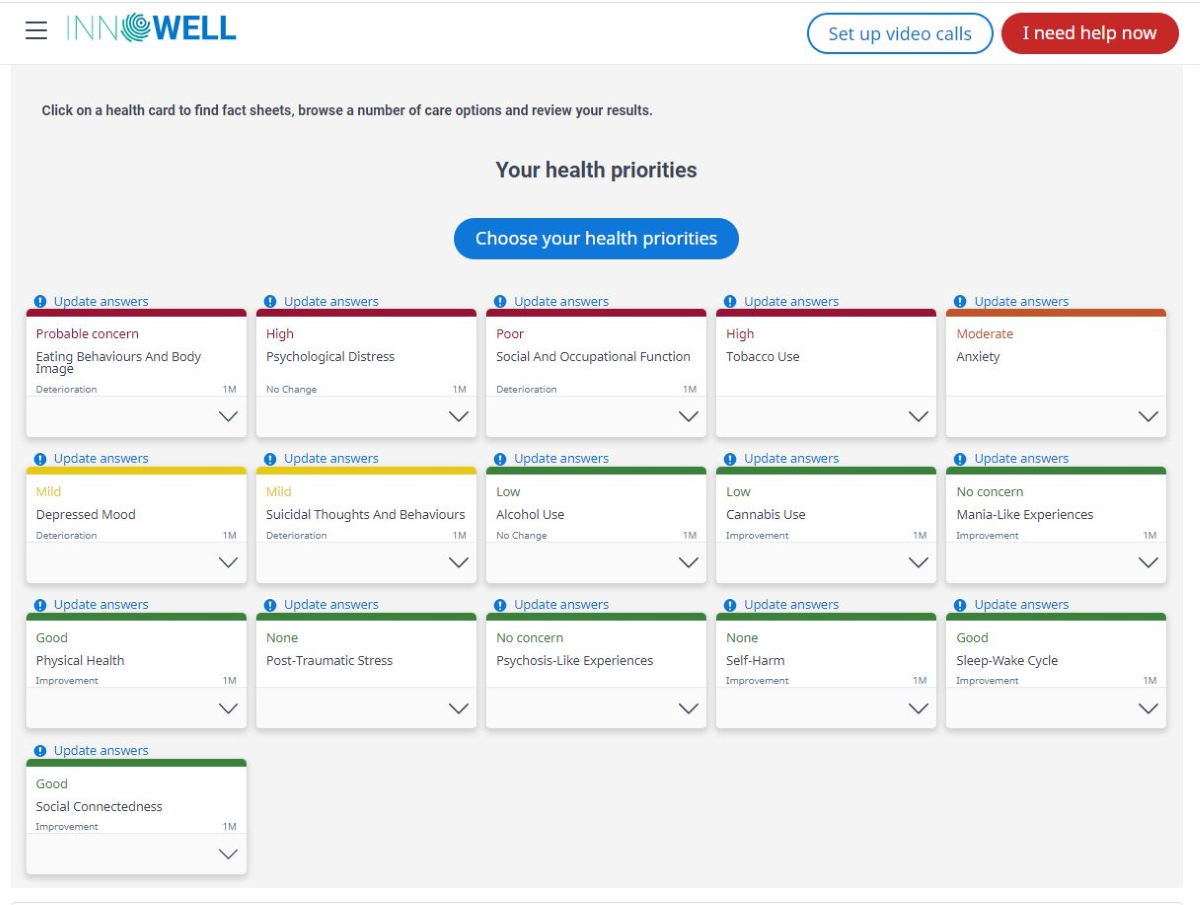
Example dashboard from the InnoWell Platform.

**Figure 2:**
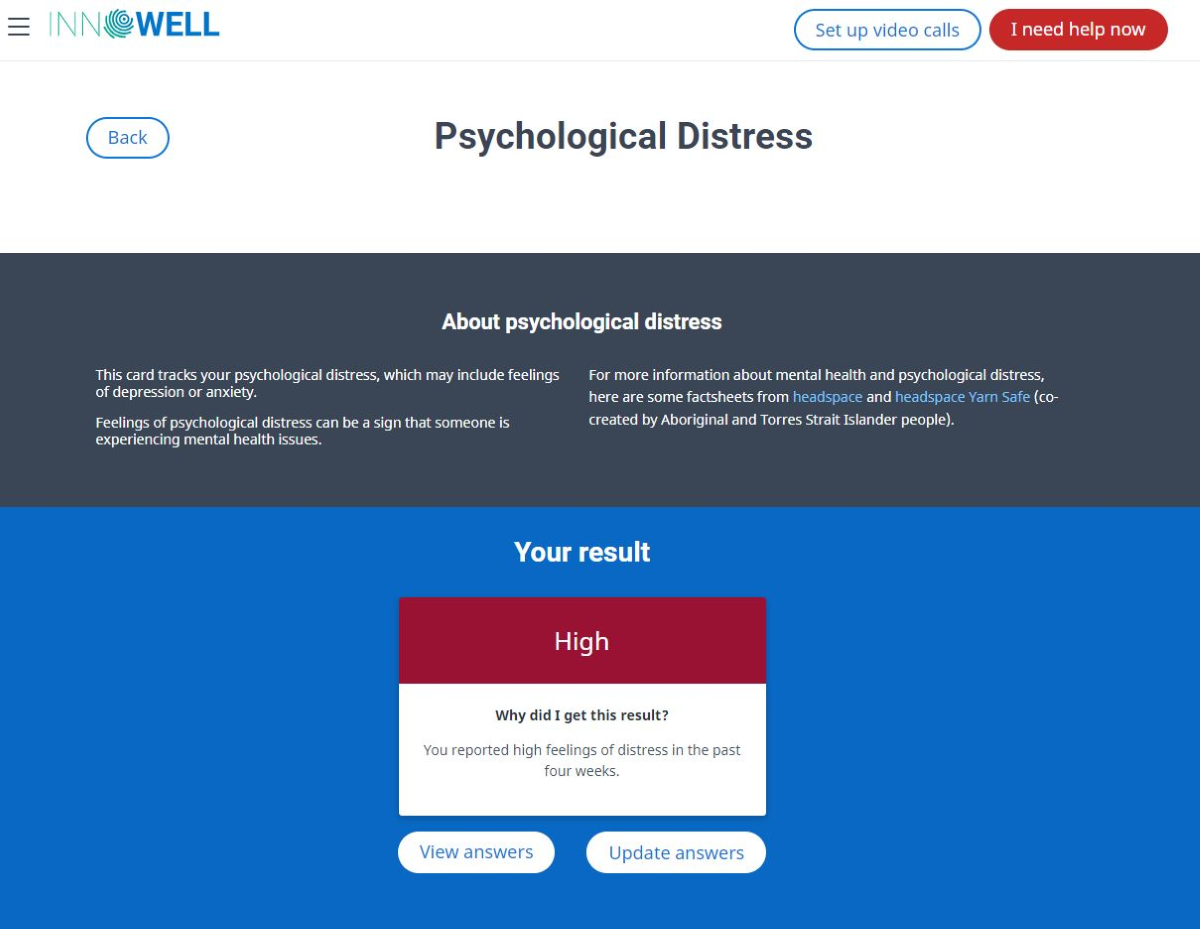
Example explanation of a health domain and assessment result from the InnoWell Platform.

As detailed by Davenport et al [20], the InnoWell Platform collects both multidimensional self-report and informant (eg, supportive others and health professionals) information via questionnaires to capture a holistic understanding of a service user’s clinical presentation, level of need, and personal preferences, to then monitor their progress over time. Specifically, survey data were collected across a range of biopsychosocial health domains, such as psychological distress, depressed mood, sleep-wake cycle, physical health, and social and occupational functioning. Further objective behavioral data can also be collected via third-party integrations (eg, Fitbit fitness trackers [Google LLC]). As shown in Figure 1, the multidimensional assessment results are presented back to service users in a dashboard color coded with a traffic light system to ease interpretability (ie, green, yellow, orange, and red are used to denote increasing degrees of symptom severity or level of impairment for each health domain). Service users are also provided with a description of each health domain and a brief explanation of the result they received (Figure 2).

**Figure 3:**
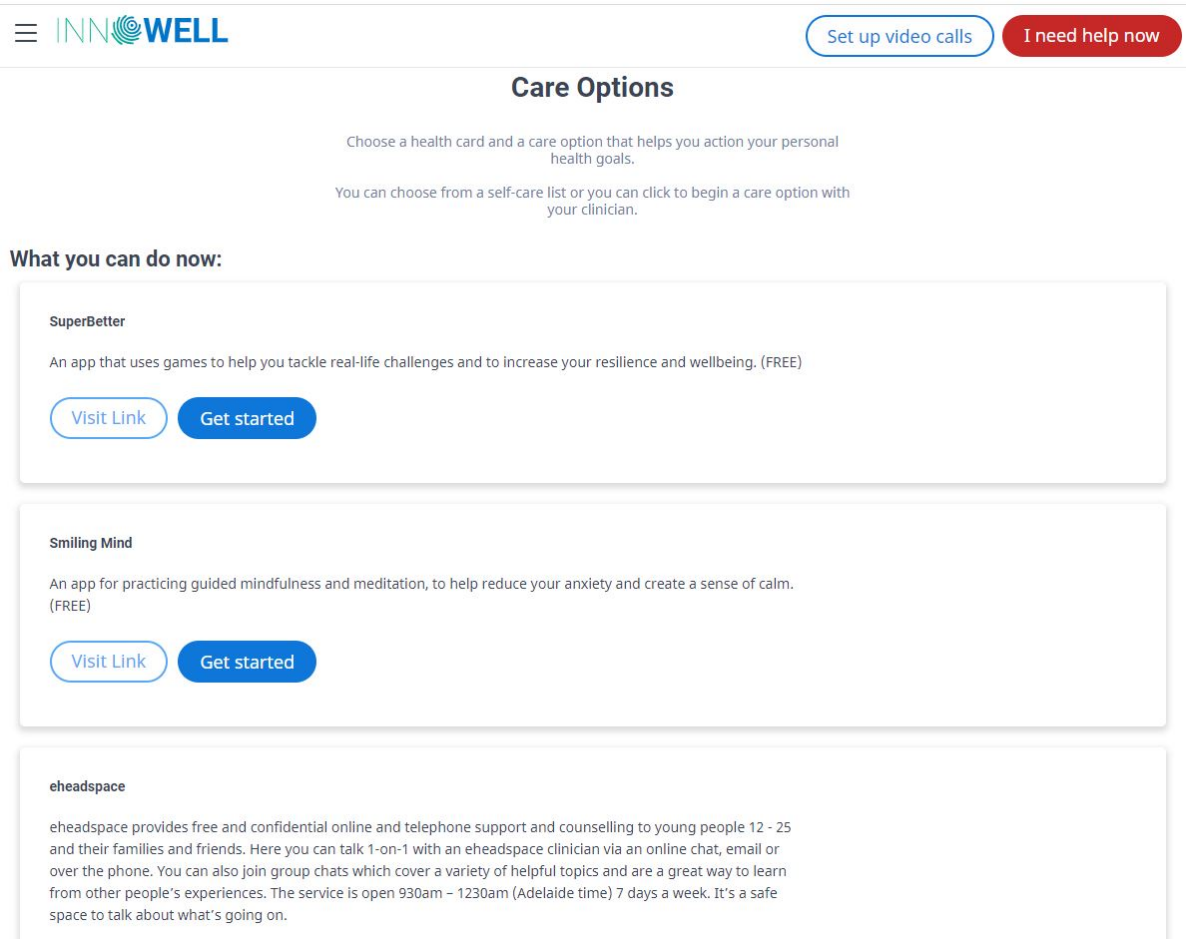
Example nonclinical care options from the InnoWell Platform.

The assessment results can then be used collaboratively by the service user and their health professional to ensure both parties are informed and actively engaged in treatment decisions in a responsive way. This is designed to occur not only at the outset of the care journey but also on an ongoing basis, as service users and clinicians jointly track progress over time. Importantly, as shown in Figure 3, service users can also access recommended nonclinical care options (eg, fact sheets, apps, e-tools, and other web-based systems) to support their mental health and well-being proactively (eg, before receiving and in conjunction with the clinical care received through the service).

### Objective

The InnoWell Platform was co-designed and implemented in diverse service settings across different age groups and populations. The Project Synergy effectiveness trials aimed to deliver critical insights about how HITs, exemplified by the InnoWell Platform, can be optimally configured and implemented as part of standard care to meet the needs of the populations under focus, their immediate care team (health professionals and service providers), and supportive others. Furthermore, a mixed methods approach was established to evaluate the web-based platform and the impact of its implementation (where available) in the individual services to iteratively refine the InnoWell Platform and the technology-enabled service model into which it was embedded.

**Figure 4:**
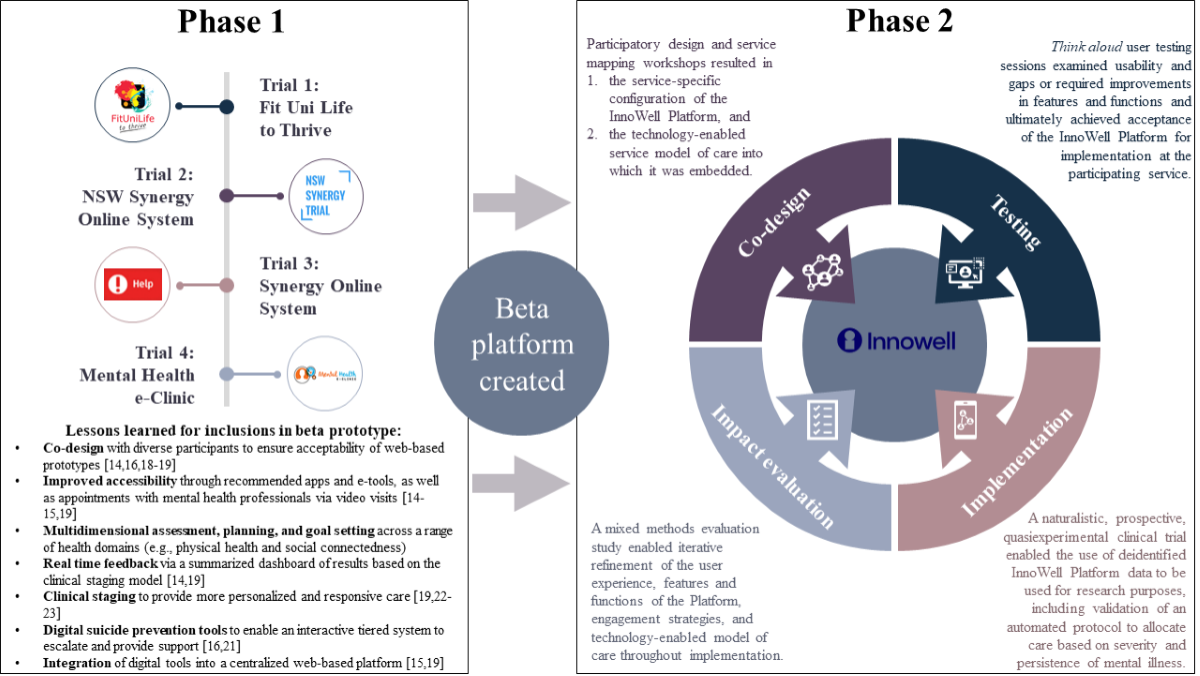
A summary of the methodologies and key outcomes from phase II of Project Synergy [14-16,18-19,21-22,23]. NSW: New South Wales.

As shown in Figure 4 [14-16,18-19,21-22,23], this paper seeks to summarize the methodologies used in phase II of Project Synergy and highlight their associated research outcomes. Specifically, we describe the process by which the original beta version of the InnoWell Platform was (1) iteratively co-designed, developed, and refined through participatory design workshops with individuals with lived experience of mental ill-health, health professionals, and service administrators to create service-specific configurations of the digital platform; (2) tested by end users to determine acceptance for each participating service or service user group; (3) implemented within a technology-enabled service model that had been co-designed with service staff through service mapping; and (4) evaluated to determine its impact at the level of the service user, health professional, and service. We provide an overview of the key outcomes from this research, highlight tools that can be used to drive the uptake of HITs by both service users and health professionals, and provide examples of how smart, digital technologies can drive innovation in models of care at the service and system level to better address demand management, early intervention, and rapid assessment and triage of at-risk users to specialized care.

## Methods

### Overview of the Project Synergy Research Streams

Phase II of Project Synergy aimed to conduct 4 rolling research streams to evaluate the engagement with and efficiency and effectiveness of the InnoWell Platform. The research streams focused on four populations across the life span, namely, veterans and their families, young children and their families, young people, and older adults. Importantly, through the internal R&D process, these rolling research streams enabled the iterative development and redevelopment, evaluation, and refinement of essential functionality within the InnoWell Platform (eg, assessment, dashboard, algorithms, real time data tracking, aggregate service indicator dashboard, immediate *staged care* recommendations, and service pathways) over time. One or more research studies were planned in relation to each research stream, in collaboration with relevant mental health services when feasible or with specific participant groups (ie, young children and families). In relation to the former, potential participating services were identified based on the alignment between their service user group or groups and the research streams. After agreeing to participate in Project Synergy, a research agreement was formalized between the University of Sydney and the participating service. As described in this *Methods* section, the methodology that underpins Project Synergy’s research trials includes co-design activities (ie, participatory design workshops, user acceptance testing, and service mapping sessions), implementation science, and a mixed methods evaluation of the impact of the InnoWell Platform and the associated technology-enabled model of care at the level of the individual, health professional, and service based on key performance indicators.

### Participating Services and Service User Groups

The InnoWell Platform was co-designed with and tested by the target end users, including individuals seeking or engaged in mental health care. Digital health solutions are widely recognized as a potential means by which to address issues of demand management in mental health globally, and, to a degree, the COVID-19 pandemic fast-tracked the uptake of technology as part of mental health care. However, it is critical to recognize that factors such as age, technical skills, literacy, and health literacy affect the ways in which individuals engage with technology [24]. Despite the proliferation of digital health solutions, such as apps, in recent years, little attention has been paid to how these tools will be used by individuals from diverse populations [25]. In addition, only 30% of apps developed by established digital health companies have tested their apps with individuals with clinical conditions [26]. To ensure that the InnoWell Platform and technology-enabled model of care met the needs of the target end users, it was crucial to ensure that the research studies were conducted with individuals engaged in clinical care. Participating services and the service users to whom they provide care are presented in Textbox 1.

**Participating mental health services and their respective service user groups.**

- Open Arms–Veterans & Families Counselling Sydney and Lismore, New South Wales (NSW), providing care to current and ex-serving military personnel and their families
- NSW North Coast *headspace* centers (Port Macquarie, Coffs Harbour, Grafton, Lismore, and Tweed Heads) providing mental health care to young people in regional NSW aged 12-25 years
- The Butterfly Foundation’s National Helpline supporting those with eating disorders and negative body image via telephone, email, and web-based chat
- Kildare Road Medical Centre—a large general practice in Blacktown, NSW, with a socioeconomically and culturally diverse service user population, including a large number of Aboriginal and Torres Strait Islander service users
- Connect to Wellbeing North Coast NSW (administered by Neami National)—a community-based primary intake and referral service located within the footprint of the North Coast NSW Primary Health Network. Within the Australian context, Primary Health Networks are operated by not-for-profit companies and are responsible for commissioning and coordinating health services to meet the needs of the people in a designated region

Additional co-design and preliminary evaluation research studies were conducted with specific service user groups not affiliated with a mental health service including older adults (aged ≥50 years) and children (aged ≥8 years) and families to inform future iterations of the InnoWell Platform. These participants were recruited through active research clinics at the University of Sydney’s Brain and Mind Centre as well as via nongovernmental and private organizations associated with the Brain and Mind Centre. The co-design studies were advertised via posters and postcards at the advertising site as well as via their websites and social media pages. Importantly, although a diverse grouping of services and service users participated in Project Synergy, further investigation is still required to determine the usability, acceptability, and relevance of the InnoWell Platform for individuals who are from a culturally and linguistically diverse background, identify as Aboriginal and Torres Strait Islanders, or identify as having a disability.

Finally, approximately 50 local stakeholders from the North Coast NSW Primary Health Network, including representatives from health and social policy agencies, nongovernment organizations, primary care providers, emergency services, research institutions, community groups, and, importantly, people with lived experience of suicide, contributed to the development of a system dynamics model. Using a broad systems perspective, stakeholders identified key outcomes of interest and subsequently provided feedback to iteratively refine the model.

### Ethics Approval

**Figure 5:**
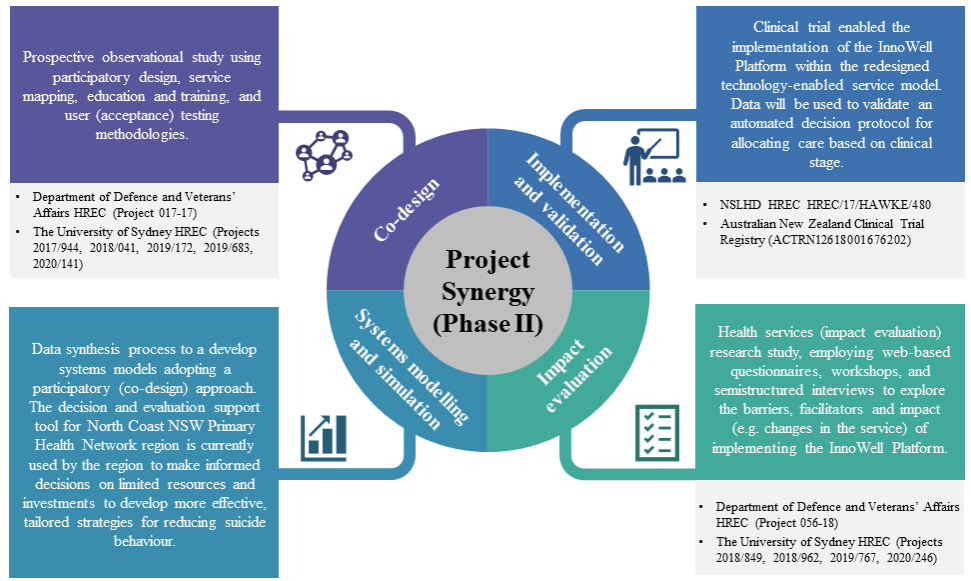
Research methodologies and ethics approvals underpinning Project Synergy. HREC: human research ethics committee; NSW: New South Wales; NSLHD: Northern Sydney Local Health District.

This research required multiple ethics approvals by various human research ethics committees (HRECs) owing to the diverse organizational structures governing each of the participating services. The clinical trial allowing users of the InnoWell Platform to share their deidentified data for research purposes was approved by the Northern Sydney Local Health District HREC (reference HREC/17/HAWKE/480) and was registered with the Australian New Zealand Clinical Trial Registry (ACTRN12618001676202). Separate ethics were sought for the co-design and impact evaluation research for each participating service. The governing bodies of some mental health services required applications to be submitted through their own internal HRECs (ie, the Department of Defence and Veterans’ Affairs HREC Projects 056-18 and 017-17), whereas others preferred that required applications were submitted through the University of Sydney HREC (Projects 2017/944, 2018/849, 2018/041, 2018/962, 2019/172, 2019/683, 2019/767, 2020/141, and 2020/246). The development of the system dynamics model did not require ethics approval as it involved secondary analysis of a broad range of aggregate data sets and synthesis of existing research. Stakeholders were invited to contribute their expertise to this model but were not the subject of the research activity. Figure 5 outlines the diverse research methodologies that were used as part of Project Synergy, underpinning all aspects of the project from co-design and development of the InnoWell Platform through to impact evaluation and dissemination of outcomes.

### Co-design

An R&D cycle, described by Davenport et al [17], was established and used to explicitly position end users as empowered participants in all stages of the research—from planning and design through to implementation and evaluation. Central to the R&D cycle are participatory design workshops to discover, prototype, and evaluate potential design solutions through the use of prompted discussion, review of working prototypes (wireframes) and existing InnoWell Platform functionality, personas (ie, a fictitious potential user for whom the product is being designed), and user journeys. Direct engagement with potential end users as well as other key stakeholders (ie, representatives from funding agencies) through targeted participatory workshops is essential to ensure the development and build of acceptable, usable, and scalable digital tools. The primary objective of the workshops was to co-design and configure the InnoWell Platform for each participating service or user group to ensure that it met the needs of the target end users, including people with lived experience accessing care and their supportive others as well as health professionals, service managers, and administrators.

Workshop agendas were tailored to each service by the research management team, comprising researchers, health professionals, and service administrators, to determine how best to discover how the InnoWell Platform might enhance standard care. Areas of focus included increased accessibility to care, waitlist management, risk identification, coordination of care with other services, and the potential for digital tools to support self-management and well-being. Participants worked together to create mockups of the technology, including the landing page, assessments, and dashboard, highlighting key features and functions that they thought would address identified *pain points* for the service and service users. Only after having captured participants’ unbiased ideas were they then provided with paper wireframes or screenshots of the InnoWell Platform on which to provide specific feedback on prototyped or existing features and functionality. The final outcome of the workshops was a service-specific configuration of the InnoWell Platform.

Importantly, the service-specific version of the InnoWell Platform was then tested by end users in one-to-one, face-to-face, 90-minute user testing sessions. Each session was facilitated by a member of the research team, and a scribe was present to take notes to capture all feedback accurately. The aim of the user testing sessions was to (1) learn if participants were able to navigate the InnoWell Platform intuitively, (2) identify any features of the platform that the user would want to see changed before implementation of the platform into the service (ie, showstoppers), (3) gain insight into participants’ experience of using the platform, (4) identify possibilities for improving the platform to better meet user needs and wants, and (5) learn how best to implement the platform into the participating service. Testers were observed interacting with the InnoWell Platform and asked to *think aloud* when exploring its features, providing both positive and negative feedback about the user experience. In addition to freely exploring the features and functionality of the InnoWell Platform, testers were also asked to complete specific scenarios relevant to their role (eg, service user, health professional, and service manager). For example, a health professional might be asked to try entering data into the InnoWell Platform about a service user and then to comment on their experience (ie, ease of completing the action and gaps in the functionality). The feedback was integrated back into the iterative R&D cycle to inform the ongoing refinement of the product by our collaborators at InnoWell. Furthermore, user testing was also used to achieve *acceptance* of the technology, signally that it was ready for implementation within the service.

Finally, in parallel with the participatory design workshops and user testing sessions, our team also used service mapping, a process mapping methodology, to understand how best to integrate the InnoWell Platform within participating mental health services. As published in LaMonica et al [27], we have used the term *service mapping* to refer to the structured approach to understanding the needs, existing processes, gaps in care, and performance of a mental health service mapped against an individual’s journey through the service. More specifically, participants worked collaboratively with the researchers to first map the current service user journey through the participating service on a whiteboard or butcher’s paper. The gaps or limitations of the current care pathway were reviewed relative to the key features of high-quality mental health care to identify areas for improvement or reform. The existing map was then revised to include technical element supported by the InnoWell Platform (eg, web-based initial assessment and clinical and nonclinical care options). The outcome was a co-designed technology-enabled model of care highlighting the intersection and impact of the InnoWell Platform at key points in care (eg, referral, assessment, treatment planning, and routine outcome monitoring).

Table 1 summarizes the research aims and co-design activities for each of our participating services and service user groups.

**Table 1:** Summary of Project Synergy co-design activities.

Population	Service partner	Research aim	Co-design activities
Current and ex-serving Australian Defence Force personnel and their families	Open Arms–Veterans & Families Counselling Sydney and Lismore, NSW^a^	Co-design, develop, implement, and evaluate the InnoWell Platform to achieve improved outcomes	4 participatory design workshops (n=21)^b^ 2 service mapping workshops (n=21) 18 user testing sessions
Young people aged 12-25 years residing in the NSW North Coast	NSW North Coast *headspace* centers (Port Macquarie, Coffs Harbour, Grafton, Lismore, and Tweed Heads)	Enhance quality and timeliness of mental health services for NSW North Coast youth	11 participatory design workshops (n=48) 9 service mapping workshops (n=46) 30 user testing sessions
Australians with eating disorders and body image issues	The Butterfly Foundation’s National Helpline	Evaluate, prototype, and configure a digital system for a nonconventional (web-based, phone, and email) service providing support for eating disorders and body image issues through the use of co-design processes	6 participatory design workshops (n=47) 2 service mapping workshops (n=12) 3 user testing sessions
NSW North Coast residents	Connect to Wellbeing North Coast NSW (administered by Neami National)	Configure and implement the InnoWell Platform to support community-based primary intake and referrals across the North Coast NSW Primary Health Network	1 user testing sessionc
Service users in a community general practice setting	Kildare Road Medical Centre, Blacktown, NSW	Configure and implement the InnoWell Platform to support Kildare Road Medical Centre’s delivery of mental health care	2 service mapping workshops (n=6)c
Older adults (aged ≥50 years)	N/A^d^	Co-design and configure the InnoWell Platform to evaluate the accessibility, engagement, and appropriateness of the technology for an older age group	4 participatory design workshops (n=21) 19 older adults consented to provide user feedback on the InnoWell Platform after using it for up to 90 days
Younger children and families	N/A	Co-design a configuration of the InnoWell Platform that is appropriate for younger children and their families	3 participatory design workshops (n=15)

^a^NSW: New South Wales.

^b^A total of 9 cofacilitated participatory design workshops, including 2 service mapping workshops, were conducted in 2017 with current and former military personnel and their families as well as Open Arms’ health professionals, service managers, and administrators from Sydney, Canberra, Maitland, Singleton, and Port Stephens [28]. Importantly, the outcomes from this collaboration served as the basis for the configuration of the InnoWell Platform with supplementary participatory workshops being conducted in 2018 specifically as part of Project Synergy.

^c^Further information informing the service-specific configurations and user acceptance of the InnoWell Platform was captured during routine project management meetings with each of the participating services.

^d^N/A: not applicable.

### Implementation and the Clinical Trial

The effectiveness of the InnoWell Platform is being assessed in a naturalistic, prospective, quasiexperimental (ie, uncontrolled, nonrandomized) clinical trial. It is important to note that this methodology is rapidly gaining recognition as a practical and efficient method of assessing change over time in users under ecologically valid conditions (ie, real-world conditions of constantly changing apps and technologies) [29,30]. Importantly, by conducting group comparisons based on clinical stage, demographics, and locality, it is possible to infer strong evidence for the usefulness (or lack thereof) of the technology-enabled blended model of care.

Regarding implementation and conduct of the clinical trial, the co-designed and configured InnoWell Platform was implemented at each of the participating services within the redesigned, technology-enabled service model as part of standard practice in accordance with our established implementation science strategy [31]. Importantly, standard practice was defined by the service; in other words, participating services had the opportunity to implement the InnoWell Platform in varying ways depending on what best suited their service and their service user base. All service users aged ≥14 years who presented to a participating service were eligible to use the InnoWell Platform as part of their care. Upon creating an account in the InnoWell Platform, users were presented with a *Terms of Use* agreement informing them that their deidentified data collected by the InnoWell Platform would be used for research purposes unless they *opt out* [32]. In 2007, the Australian National Statement of Ethical Conduct in Human Research was updated to enable HRECs to approve an *opt-out* consent for low-risk clinical research [33]. *Opt-out* approaches require researchers to ensure that participants are aware of their potential inclusion in research and given the opportunity to indicate that they do not want their data used in this way (ie, thus opting out). Importantly, service users were able to update their data sharing permissions in the InnoWell Platform at any time, enabling them to, for example, withdraw from the clinical trial should they wish to do so. As the InnoWell Platform is an exempt medical device, it is not required to be included in the Australian Register of Therapeutic Goods and can be used by service users regardless of whether they opt out of the clinical trial.

In addition to the sharing of data for research purposes, service users are also informed that their data will be shared with the participating service at which they are receiving care. This information is made explicit to the user at the point at which the InnoWell Platform is originally explained to them by a staff member from the participating service using a simple script delivered via telephone, email, chat, or in person, and in the subsequent email invitation they receive inviting them to use the InnoWell Platform. In addition, this information is documented in the InnoWell *Privacy Policy*, which is available on the landing page, before creating an account and providing any personal information.

The primary objective of the above-mentioned health services trial is to validate an automated decision protocol for allocating care based on severity and persistence of mental health symptoms (ie, based on the clinical stage) [34] relative to the clinical stage as allocated by health professionals, multidisciplinary teams via consensus, and an expert clinical reference group [20]. The protocol aims to automate critical decision points to triage service users based on clinical stage. Specifically, the first decision determines whether there is clear evidence of at least one full-threshold, major discrete disorder, or a persistent and recurrent syndrome or syndromes, whereas the second decision aims to determine whether the syndrome is nonspecific or attenuated. By identifying the clinical stage and level of need, service users can then be automatically triaged to self-management strategies including self-directed apps and e-tools, ambulatory care services, or acute or specialized services at the point of service entry. As detailed by Sawrikar et al [35], clinical staging avoids the *fail first* approach typified by stepped care in favor of allocating the *Right care, First time*. The clinical trial allows for the collection of deidentified data for research purposes, and cross-sectional findings from the participating services are provided in Multimedia Appendix 1 [36-40]; however, the findings from the clinical trial are beyond the scope of this paper and will be published separately.

An automated suicide escalation protocol is embedded within the InnoWell Platform, designed to identify service users reporting suicidal thoughts or behaviors and, importantly, suggest pathways to appropriate and timely care [21]. Specifically, when a service user expresses suicidal thoughts or behaviors on the InnoWell Platform’s multidimensional assessment, they are immediately provided with a pop-up message containing contact information for relevant 24-hour crisis support services (ie, Lifeline and Kids Helpline). In addition, to enable a rapid clinical response, a notification is also sent to the clinical service when the degree of risk meets a predetermined severity threshold established by the service for their service user base.

### Impact Evaluation

Traditional clinical science approaches to the development and implementation of interventions rely on a lengthy linear process, from basic science through to effectiveness trials and dissemination [41], which can result in delays of up to 17 years for research translation into clinical practice [42]. Given the rapid advances that occur in the technology sector, this is not a practical approach to the evaluation of HITs or technology-enabled models of care. To that end, Mohr et al [29] highlight the benefits of evaluating the challenges and successes associated with tools such as the InnoWell Platform in targeted clinical settings, such as through the Project Synergy participating services, to iteratively refine and optimize usability, acceptability, and effectiveness to ensure that it meets identified clinical objectives. Therefore, as detailed previously by LaMonica et al [10], we used a mixed methods impact evaluation study, including web-based surveys, semistructured interviews, and workshops, to identify potential barriers to and facilitators of implementation and to evaluate the impact of the InnoWell Platform on the participating mental health services over time, including (1) digital literacy and competence of the service staff in relation to the implementation of the HIT in the service; (2) changes in the service in association with the implementation of the HIT-enabled solution; and (3) the quality, usability, and acceptability of the solution. All participating staff involved in the implementation of the InnoWell Platform at each of the respective services, including health professionals, service managers, and administrators, were invited to participate in the impact evaluation study. Where staff from the services’ funding or governing bodies (ie, service providers or primary health networks) were associated with implementation, these staff were also invited to participate and were required to provide written informed consent. The impact evaluation data were collected at baseline and every 3 months thereafter for the duration of the implementation, the length of which varied based on agreements made with participating services. Importantly, the use of mixed methods allowed the service staff to provide feedback in a manner that suited their work schedule (ie, web-based helpline operating on a 16-hour schedule vs traditional face-to-face counseling services operating during normal business hours), thus ensuring both breadth (via surveys) and depth (via the semistructured interviews and workshop) of evaluation from a broad range of participants.

### Participatory Systems Modeling

As referenced previously, a system dynamics model was developed using a participatory approach that involved approximately 50 local stakeholders, including representatives from health and social policy agencies, nongovernment organizations, primary care providers, emergency services, research institutions, community groups, and, importantly, people with lived experience of suicide [43]. Over a series of 3 workshops, stakeholders were asked to draw on the deep tacit knowledge and diverse perspectives of these system actors to map the local health system and the role that social determinants play in psychological distress and suicidal behaviors. These workshops focused on prioritization of the key outcomes of interest for the model, the mapping of pathways and drivers of those outcomes, and the prioritization of interventions to be included in the model. A full description of this process, the model, and its results can be found in the study by Occhipinti et al [43].

## Results

### Co-design

The results of most of our team’s co-design work have been published previously and will therefore not be presented in detail [27,28,36-38,44]. However, a summary of the key insights derived from the participatory design workshops is provided in Table 2, including findings regarding the (1) support for HITs as part of clinical care, (2) critical factors to consider in relation to the design and content of HITs, and (3) potential for incongruity between health professional and service manager attitudes and beliefs toward HITs during co-design relative to implementation.

In addition to the participatory design outcomes, service mapping also proved an effective way to engage key stakeholders in reflecting on existing service and system pathways and, in turn, identifying current barriers to and facilitators of quality mental health care. As highlighted in LaMonica et al [27], by working collaboratively with participating services, service mapping highlighted key gaps in care that might be improved through the implementation of the InnoWell Platform, while also identifying aspects of care that could be improved through changes to specific service-level processes (ie, intake). In this way, the service mapping process prevented a *one-size-fits-all* approach to service reform, ensuring that specific contextual factors impacting the quality of care at each service were considered, thus optimizing the service delivery. Each participating service was able to determine how best to integrate the InnoWell Platform as part of standard care as it pertained to the respective services. For example, at Open Arms and most *headspace* services, service users were invited to complete the InnoWell Platform’s multidimensional assessment before their first appointment, whereas at Kildare Road Medical Centre (KRMC), a general practice, service users were only invited to the InnoWell Platform after having an appointment with a mental health nurse who determined whether the digital tool was appropriate for their individual care needs. Importantly, our research shows that the process of mapping technology-enabled service models can lead to improvements in service delivery pathways, both in relation to and independent of the technology [28].

**Table 2:** Key insights learned through participatory design workshops.

Key insights	Specific findings
Overwhelming support for integrating HITs^a^ into mental health care across populations and service settings [4,27,28,36-38,44].	The results of a lived-experience-led national community consultation program showed considerable service user interest in and experience with digital health solutions. On the basis of data collected through 5 digital engagements, 8 face-to-face consultations, and 2 community engagement events, 81% of respondents indicated they were comfortable sharing mental health experiences on the web and 94% had already done so [45].
Service users and carers further emphasized the importance of being provided reputable (academic, government, or nonprofit mental health organization) care options and information [37].	The results of 4 participatory design workshops conducted with 21 community dwelling older adults (aged ≥50 years), including carers, highlighted the need for information delivered via HITs to come from a credible source to be perceived as trustworthy and reliable [37].
Concerns with data privacy and security of personal and health information were prevalent across all service user groups [28,37,46]; however, health professionals questioned whether all users would be wary of security risks.	Young people emphasized the need for privacy information to be readily available to allow a user to be completely comfortable when entering sensitive information into a HIT (“Always ask, could this site be more secure with my information.” [Member of the headspace Youth Reference Group, participatory design workshop in Wollongong, August 30, 2018]). Given the ubiquity of technology use by young people, some health professionals questioned whether they would be suitably concerned about sharing personal data (“Young people may be so used to this as they have grown up with it that they would not see sharing personal data as a major barrier to accessing a health and wellbeing e-tool” [Health Professional, participatory design workshop in Coffs Harbour, December 4, 2018]). Members of the military community emphasized the need to be fully transparent in relation to limitations to confidentiality and data sharing (“Data security needs to be highlighted, particularly that the information is not shared with DVA [Department of Veterans’ Affairs].” [Veteran, participatory design workshop, August 24, 2017]) [28]. Older adults indicated that data privacy and security risks are a primary barrier to the use of HITs (“Anything on the Internet I just don’t really trust, I don’t want to put my information of any kind out there.” [Older Adult, participatory design workshop in Sydney October 9, 2019]) [37].
There is a gap in what is expressed during the co-design process and actual implementation; often, clinicians are very active and willing supporters during the co-design process, however not in practice [47].	The 48 participants, including young people, supportive others, health professionals, service managers, and administrators, from 10 participatory design workshops with headspace services in the North Coast PHNb, recognized the potential for HITs to improve service quality and efficiency; however, a qualitative review of 70 fortnightly logs completed by on-the-ground implementation officers working across 5 headspace centers revealed persistent resistance to change [47].

^a^HIT: health information technology.

^b^PHN: Primary Health Network.

### Implementation and the Clinical Trial

#### Overview

To address known barriers, as well as those that might be specific to individual services, the R&D team developed a protocol for the implementation of digital solutions into mental health services, including digital tools and technology-enabled models of care [31]. The protocol supports active collaboration between the researchers, health services, and service providers and users and is intended to promote mitigation strategies that might enable the successful translation of new innovations into standard clinical practice. This not only includes a strong emphasis on co-design but also acknowledges that a well-designed technology product is not sufficient to drive improved outcomes or mental health reform. Therefore, the protocol highlights the need to (1) upskill the mental health workforce regarding the evidence-based digital, clinical, service, and safety elements essential to quality mental health care, as well as how digital solutions can support the delivery of such care; (2) provide on-the-ground support to both service users and staff in the early stages of implementation to troubleshoot any issues with the technology and to help interpret and report clinical data in a meaningful way; and (3) refine iteratively the digital solution, as well as the technology-enabled model of care, in response to service-level changes in outcomes and processes or user feedback on the quality, usability, and acceptability of the solution. In relation to the latter, it is crucial to continue to refine digital solutions to ensure they become an integral part of standard care, enabling improved outcomes on key service performance indicators. Concurrently, minimizing increased burden on service staff associated with the implementation is challenging. However, striking this balance may afford the best opportunity for the sustainability and scalability of the solution.

The naturalistic, quasiexperimental clinical trial to validate the InnoWell Platform is in progress. At the time of this publication, 1644 service users aged ≥14 years consented to share their data for research purposes. Cross-sectional data from the initial multidimensional assessment for each participating service are provided in Multimedia Appendix 1. Further analysis of the data collected by the InnoWell Platform will be conducted at the service-specific level and is beyond the scope of this paper. Further, the data to validate the automated decision protocol for allocating care based on clinical stage will be analyzed and published separately. Importantly, however, the automated suicide escalation protocol embedded within the InnoWell Platform has been shown to effectively identify service users requiring urgent risk assessment. For example, 22.6% (24/106) of the young people using the InnoWell Platform as part of their care at a *headspace* service in the regional North Coast NSW Primary Health Network footprint reported high levels of suicidal thoughts and behaviors (STBs), thus triggering an immediate notification to the service. A similar proportion (18/62, 29%) of users from Open Arms underwent a rapid risk assessment after the service received a notification of moderate to high levels of STB as self-reported via the assessments in the InnoWell Platform. Although the management of such notifications can be challenging, services recognized the need for this functionality, as it fast-tracks care.

#### Innovative Models of Enhanced Primary Care

The implementation science strategies resulted in an innovative model of enhanced primary care, best suited to local implementation. Specifically, KRMC is a large Western Sydney family practice with 26 general practitioners (GPs) seeing an average of 25 to 30 service users per day. As part of their engagement in Project Synergy, KRMC used a mental health nurse to support their GPs with assessment and triage, treatment planning and intervention, and case review of those who presented with symptoms suggesting mental ill-health. The mental health nurse was a critical facilitator of the co-designed technology-enabled service delivery model at KRMC. For example, the application of the InnoWell Platform’s suicide escalation protocol [21] identified 13% (17/131) of those service users as having STBs, thus enabling the mental health nurse to triage these individuals rapidly to appropriate care. The mental health nurse also reviewed the initial assessment results in collaboration with each service user to guide a discussion about available care options, considering service user preferences. Importantly for KRMC, the technology-enabled model of care, as led by the mental health nurse, resulted in expanded treatment options, including the availability of brief psychological therapies conducted by the mental health nurse as well as the apps and e-tools embedded within the InnoWell Platform. Furthermore, the mental health nurse was also able to use the multidimensional assessment to draft a mental health care plan for later review by the GP. Consequently, this reduced the assessment time required by the GP, allowing them to focus instead on delivering *value-added* care to more service users. In light of the success of this pilot study, KRMC sought funding to support the ongoing implementation of this innovative technology-enabled, mental health nurse-led service delivery model.

#### An Innovative Model of Enhanced Community-Based Assessment and Triage

**Figure 6:**
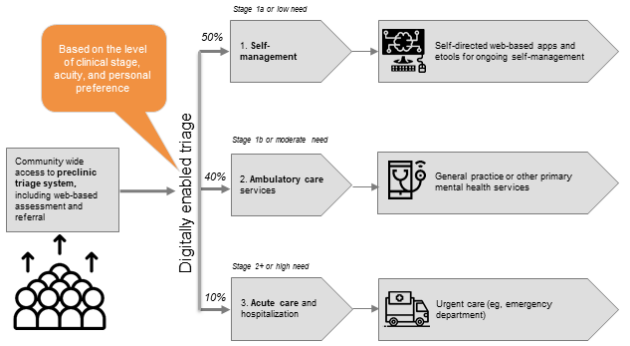
Preclinic triage system enabled by the InnoWell Platform (as originally published by Davenport et al [45]).

As previously reported [39], the R&D team partnered with Neami National (who deliver the central community-based intake service of the NSW North Coast Primary Health Network—*Connect to Wellbeing North Coast*) to co-design and implement a new technology-enabled assessment and triage system for the range of services offered [39]. Specifically, the InnoWell Platform’s multidimensional assessments were used to (1) identify service users with risk factors and symptoms or impairment (stage 1a) who were immediately triaged to self-management including self-directed and clinician-supported apps and e-tools, (2) assist those with attenuated or subthreshold major disorders (stage 1b+) to clinical care within ambulatory care services including general practice or another primary care service, and (3) provide immediate clinical assessment for those with a risk of self-harm and determine the need for acute care or hospitalization. This model could serve as a prototype for a direct-to-service user preclinic triage system (Figure 6). Such a system is scalable and directly addresses the Productivity Commission’s call to develop a sustainable national digital platform to facilitate assessment and referral processes that match levels of mental health care more directly with service users’ actual needs [3].

### Impact Evaluation

As shown in Table 3, data from the impact evaluation results highlight consistent agreement regarding the potential for HITs to improve outcomes for service users and mental health services alike. However, participants, including health professionals, service managers, and administrators, indicated that technologies are generally not well integrated into current service delivery models. Service readiness for change (eg, existing technology infrastructure and the digital literacy of staff and service users) was noted to be a potential barrier to successful implementation, with less than half of respondents indicating that their service was ready to implement new technologies to enhance mental health care. Furthermore, there was considerable variability among clinical staff as to whether it was their responsibility to recommend technology as part of standard care.

The impact evaluation findings were used to iteratively refine the technology-enabled model of care throughout the implementation of the InnoWell Platform at each participating service. For example, based on findings from the evaluation activities, services modified (1) the point in the care journey at which the InnoWell Platform was offered to participants, (2) the required qualifications for the person presenting the platform to service users for the first time (eg, mental health nurse, counselor, or clinical psychologist rather than administrative staff), (3) the technology available to health professionals at the service to be able to best use the InnoWell Platform actively during sessions, and (4) processes by which the data collected by the InnoWell Platform were used to inform care. Furthermore, the findings were fed back to service leadership to help find ways to better support health professionals and administrative staff to use the InnoWell Platform effectively and efficiently as part of standard care. Finally, feedback regarding the usability and acceptability of the InnoWell Platform as well as identified gaps or limitations in its features and functionality were fed back to InnoWell as part of the internal R&D cycle to inform the development or redevelopment and refinement of the technology.

**Table 3:** Impact evaluation outcomes.

Key outcome	Specific findings
Staff across multiple service settings consistently support the use of technology as part of their work; however, they also list digital literacy of both service users and health professionals as well as service readiness for change as potential barriers to widespread adoption [10].	A total of 81% (38/47) of health professionals and service administrators reported benefits of using technology as part of their work. Most staff (26/45, 57%) questioned whether their service users’ digital literacy was sufficient to use technology as part of their mental health care; however, of potential users, young people, who were considered digital natives, were expected to be most likely to access and adopt technologies as part of care. Although approximately two-thirds (27/45, 60%) of staff indicated that their service’s policies support the belief that technologies can improve service user outcomes by providing more efficient and effective care, only 44% (20/45) of service staff indicated that their service was ready to implement new technologies to enhance mental health care. Furthermore, only 53% (24/45) of staff reported that their service actively encourages the integration of technologies as part of standard care.
There must be organizational leadership (PHN^a^-level decision-making) as well as a local champion at the service level to support a successful implementation [4].	Qualitative feedback was collected from 40 staff from across 5 headspace centers in the Central Eastern PHN who were involved in implementing a prototype of a web-based mental health clinic, known as the Mental Health eClinic. Their feedback highlighted the significant benefit of both (1) high-level endorsement and coordination at the PHN level and 2) the presence and engagement of on-the-ground leadership to assist in solving day-to-day implementation challenges [4]. Qualitative analysis of 70 logs completed by on-the-ground implementation officers at 5 headspace services implementing the InnoWell Platform showed a consistent interest among service staff in implementing the InnoWell Platform as a demand management tool and to provide better care; however, a lack of strong and deliberate leadership was highlighted as a barrier to change. Notably, this finding did not change appreciably in response to COVID-19 [47]. To facilitate a successful implementation, service leadership need to establish clear strategies to mitigate potential barriers to implementation identified by on-the-ground staff responsible for driving the implementation [10].
Health professionals are often confident about the effectiveness of their current service models (ie, business as usual) and express reluctance to change their usual practices [10].	A primary implementation barrier identified by health professionals relates to concerns that digital tools could replace clinical expertise; however, all participants denied this as a personal concern.

^a^PHN: Primary Health Network.

### Participatory Systems Modeling of the North Coast Primary Health Network Mental Health Services

The R&D team was engaged to work with the North Coast Primary Health Network to model the mental health services available across the region and test the relative impacts of a range of interventions on mental health and prevention of suicide. This work was critical for Project Synergy, as one of the primary interventions of interest was technology-enabled care coordination. The capacity to compare the impact of this system-level intervention with other clinical and health system alternatives was a major advance. The model was validated against historic time series data (2011-2017), and then projected population-level trajectories of suicidal behavior in the region using a 20-year time horizon (2021-2041) were derived. Against the baseline trajectory, implementation of technology-enabled care coordination was projected to decrease suicide deaths by 5.6% (95% CI 4.8%-6.5%) [43]. The model also found that the most effective combination of interventions to reduce suicidal behavior was supporting social connectedness, technology-enabled coordinated care, postsuicide attempt assertive aftercare, reductions in childhood adversity, and increasing youth employment [43]. This combination of interventions was projected to reduce self-harm hospitalizations (indicative of suicide attempts) by 28.5% (95% CI 26.3%-30.8%) and suicide deaths by 29.3% (95% CI 27.1%-31.5%) among a youth population (aged 15-24 years). Introducing additional interventions beyond this best performing suite of interventions produced only marginal improvement. That is, “more is not necessarily better” [43]. Further analysis demonstrated that technology-enabled care coordination led to greater reductions in suicide deaths, the total number of self-harm hospitalizations or mental health–related ED hospitalizations, and the prevalence of high psychological distress in the population, when accompanied by increases in service capacity growth by 20% (including standard telehealth practices [48]). Findings from these simulation models indicate the significant promise offered by HITs when effective implementation is achieved.

### Tools to Support the Adoption of Digital Solutions in Clinical Practice

As noted previously, service and health professional readiness for change are 2 key factors that impact the implementation of digital solutions. To help build confidence in the use of apps and e-tools in practice, the R&D team adapted the original Mobile App Rating Scale (MARS; a reliable and internationally recognized app rating system [49]) to ensure it is appropriate for health-related apps as well as e-tools (eg, websites and web-based courses). It is now known as the A-MARS [50]. A complimentary quality assurance protocol was also developed to guide health professionals in the evaluation of the quality and safety of health-related apps and e-tools to determine their appropriateness for use in clinical practice. This approach includes (1) a broad exploration for available apps and e-tools; (2) shortlisting of those health-related apps and e-tools deemed to match the needs of the service users, health professionals, or service; (3) evaluation using the newly developed A-MARS; and (4) review of the ratings compared with service-specific criteria to determine appropriateness for recommendation.

Given the consistent concerns regarding data security and privacy highlighted in our co-design work, the R&D team also developed a privacy risk assessment tool to assess whether current apps and e-tools are meeting privacy standards [46]. Our review revealed consistently poor readability, resulting in marked limitations in the transparency of the information presented. This, in turn, can undermine a service user’s trust in the privacy of their personal and health information. To assist health professionals and service providers in understanding potential privacy risks, we developed easy-to-use guidelines for their consideration before promoting individual apps and e-tools as part of care [46]. It is our recommendation that these guidelines be adopted to ensure that HITs are used to their full potential to maximize service user health outcomes while minimizing risk and users are informed of privacy and security considerations to be able to make educated decisions as to how they would like to share their personal and health information [46].

## Discussion

### Principal Findings

Recent enhanced funding for mental health care in Australia has been focused largely on increasing access to traditional clinic-based psychological therapy [51], with little to no emphasis on delivering quality care matched to the level of need, coordinating systems of care, or accounting for the unequal distribution of resources across urban, regional, and rural communities. Although the increased investment in mental health included in the Australian Government Federal Budget of May 2021 signals recognition of the need for system reform, there is a relative lack of funding dedicated to service innovation, health systems research, or evaluation [52]. These omissions are particularly concerning given evidence highlighting that technology-enabled care coordination has the potential to differentially improve health outcomes (ie, reductions in deaths by suicide, mental health–related hospitalizations, and rates of high psychological distress in the community) and assist with demand management over and above any impact owing to a 20% increase in service capacity and standard telehealth services [48]. Furthermore, technology-enabled coordinated care in conjunction with programs targeting the social determinants of health would appear to offer a good return on investment, particularly in regions with limited health resources [43].

**Figure 7:**
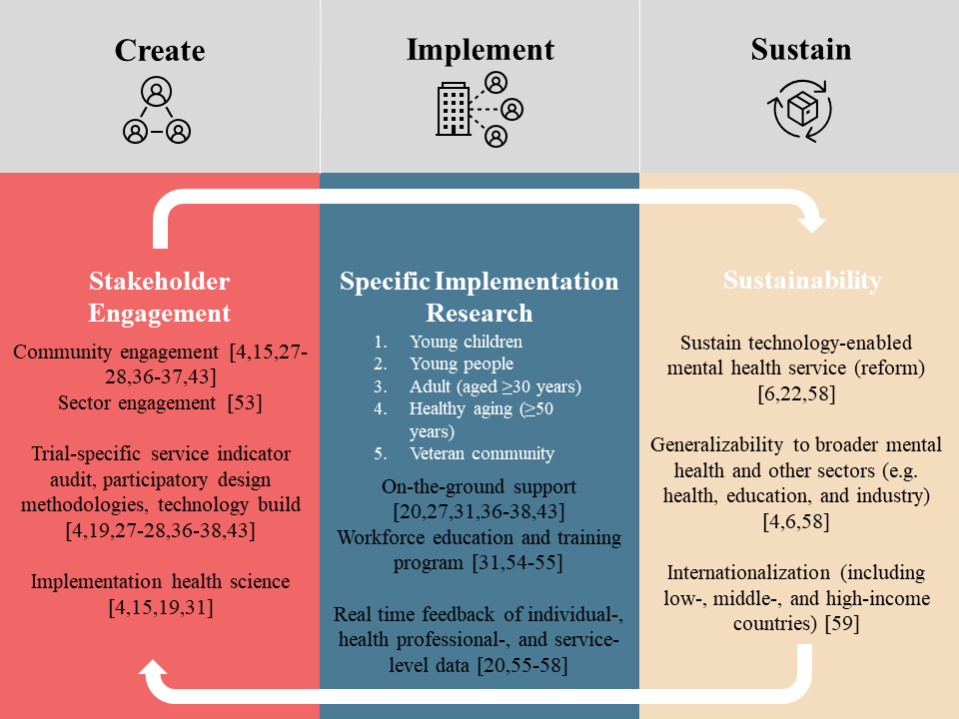
Critical learnings from Project Synergy [Figure 7 [4,6,15,19-20,22,27-28,31,36-38,43,53-59]].

With the stage set by the population-level modeling outcomes, Project Synergy has now delivered critical outcomes regarding the practicalities of designing, implementing, and evaluating digital mental health solutions (Figure 7 [4,6,15,19-20,22,27-28,31,36-38,43,53-59]). These outputs are critical pieces needed to inform delivery of effective technology-enabled and coordinated mental health care, as called for by the Productivity Commission [3] and the World Economic Forum [60]. We now have the methodologies, tools, and essential insights to build robust, dynamic, sustainable, scalable, and coordinated systems of mental health care. This has the capacity to firmly establish Australia as a leader in the delivery of digital mental health care.

On the basis of the findings from Project Synergy, it is critical to ensure that all digital solutions and innovative models of technology-enhanced systems of care be developed in collaboration with the intended target audiences, including those with lived experience of mental ill-health. However, it is important to recognize that the co-design process must also consider the needs and time demands on health professionals. Historically, health professionals have been reluctant to drive service reforms, frequently arguing instead simply for more of the same (ie, *endless therapy*) [61]. Similarly, our results highlight the potential for health professionals to be reluctant to implement digital tools as part of standard practice. To that end, research indicates that health professionals are most likely to accept changes that they are prepared for and that they value, especially those that are likely to result in direct benefits to service users [62]. Furthermore, it is essential to provide health professionals with the necessary education and training to support their understanding and use of digital health solutions. It needs to be very clear that the primary goal is to deliver high-quality and person-centered care, not simply operational or financial efficiency. Until digital health care is included as part of the core curriculum for standard health professional education, services are also likely to benefit from including a digital navigator on their care team. These new personnel can evaluate the suitability of available digital solutions for the service, troubleshoot technical difficulties, and assist in interpreting and reporting data collected via digital tools in a meaningful way to inform care and self-management strategies [63].

As existing digital infrastructure is so poorly integrated, health professionals are frequently required to enter the same data across multiple systems, including client management software, electronic medical records, minimum data sets, and personal health records. Only by reducing this administrative burden are health professionals likely to engage with new digital solutions; therefore, there is an urgent need to ensure open application program interfaces are built into all digital solutions to enable interoperability or the transfer of relevant personal and health information between various systems. Such integration can inform shared decision-making, track progress over time in coordination with and between care teams and supportive services, and empower the service user, along with families and carers, to be informed and active partners in care.

Systematically collecting multidimensional assessment data through self-report and clinical digital tools, as well as wearables and smartphone sensors, will provide the opportunity to examine service user outcomes, health professional practices, and service-level performance in relation to key indicators, including safety, acceptability, efficiency, accessibility, effectiveness, coordination, and workforce capability [64]. More specifically, assessment data enables services to understand the clinical complexity of those service users presenting for care. This can ensure that the best care options are offered immediately, as well as promoting coordination of care with other relevant specialist or support services. This type of data may also help services provide necessary training opportunities for their workforce. Furthermore, routine outcome monitoring enables data-informed decisions regarding the effectiveness or lack thereof of treatment plans to facilitate necessary changes or referrals to specialist care as needed. Finally, data linkage at the state and national levels, using data from health records (ie, Medicare Benefit Scheme and Pharmaceutical Benefits Scheme), provides the necessary information for analysis of the costs and effectiveness of various models of care. Taken together, these methods of evaluation offer an opportunity for accountability in service delivery.

Having successfully delivered the necessary building blocks to overhaul the mental health care system through Project Synergy, it is now time to translate these insights into action. With the ever-increasing demand for more personalized and higher-quality services [65], it is clear that simply increasing investment into various clinical services delivered in isolation will not be enough to improve the outcomes for the whole system; instead, new models of integrated care, and the digital infrastructure to support them, is needed to accompany these new clinical investments. Project Synergy supports calls for increased use of digital technologies in mental health care; however, it also highlights that the way these technologies are deployed really matters. Specifically, dynamic systems modeling shows that when digital technologies are limited to standard telehealth practices (ie, by extending existing services on the web), without changing the underlying model of care, the impact is low [48]. Although standard telehealth will now be implemented more widely, following the COVID-19 pandemic, to date, little effort has been made to use these technologies in ways that promote multidimensional team-based care or maximize the benefits that these technologies can really provide. By capitalizing on digital tools and investing in digital infrastructure, we now have the opportunity to recalibrate the whole mental health system, resulting in a greater impact on outcomes as opposed to those expected by simply improving the capacity across individual components of the existing mental health system.

### Conclusions

Although the Australian Government continues to invest in more clinical services, the well-documented failings of the mental health system (notably restricted and delayed access to quality care, siloed services, inadequate use of routine outcome monitoring, and care plans that do not match a service user’s level of need), may be left unaddressed. Project Synergy highlights the power of more innovative, digitally enhanced systems to increase the efficiencies of health care systems by addressing demand management, reducing delays in access to appropriate care, enabling deployment of early intervention strategies, and rapidly assisting at-risk service users to access acute and specialist care. System-level innovation can be achieved via the engagement of the appropriate service leadership and active promotion of the interoperability of the new technologies.

## Appendix

Multimedia Appendix 1 Data from the InnoWell Platform for Participating Services.

## Abbreviations

CRC: Cooperative Research Centre
GP: general practitioner
HIT: health information technology
HREC: human research ethics committee
KRMC: Kildare Road Medical Centre
MARS: Mobile App Rating Scale
NSW: New South Wales
R&D: research and development
STB: suicidal thoughts and behavior
