# Supplementary material for: Informing the Future of Integrated Digital and Clinical Mental Health Care: Synthesis of the Outcomes From Project Synergy
Source: JMIR Ment Health. 2022 Mar 9;9(3):e33060. doi: 10.2196/33060 (PMC8943544; doi:10.2196/33060)
Supplement: Multimedia Appendix 1 [file mental_v9i3e33060_app1.docx]

### Multimedia Appendix 1. Data from the InnoWell Platform for Participating Services

#### Open Arms (Sydney and Lismore) – Veterans & Families Counselling Service

The InnoWell Platform was implemented at Open Arms (Sydney) from February 2019 and Open Arms (Lismore) from August 2020, with both implementations continuing until December 2020. A total of 64 service users (40 males; 63%) completed the onboarding process. The median age was 38 years (youngest = 15; oldest = 69 years). Five (8%) service users identified as being Aboriginal. Most users were living with their family (n=44/64; 70%); however, 13 (21%) were living on their own, three (5%) lived in a share house, one (2%) reported being homeless, and one (2%) reported ‘other’ living circumstances. The majority of service users had at least completed some form of tertiary education; certificate or diploma (n=31/64; 48%), post-graduate degree (n=11/64; 17%), and undergraduate degree (n=4/64; 6%).

As shown in Figure 3, results from the multidimensional assessment indicate that the majority of service users (n=41/64, 64%) were reporting high or very high levels of psychological distress. Approximately one-third (n=20/60, 33%) of service users endorsed moderate to severe levels of alcohol use, potentially putting them at risk for health, social, financial, legal, and/or relationships problems. Additionally, one-third (n=20/59, 34%) reported experiencing high levels of anger during the previous four weeks. Nine clients (n=59, 14%) reported both excessive alcohol use and elevated levels of anger. There were 35 clients (n=59, 59%) who reported that they had experienced an unusually or especially frightening, horrible, or traumatic event, and more than half (n=16/25, 64%) of those that went on to complete the PTSD Checklist (Civilian Version) [67] scored in the high to very high range. Finally, nearly all service users (n=54/59, 92%) reported moderate to severe sleep disturbance.


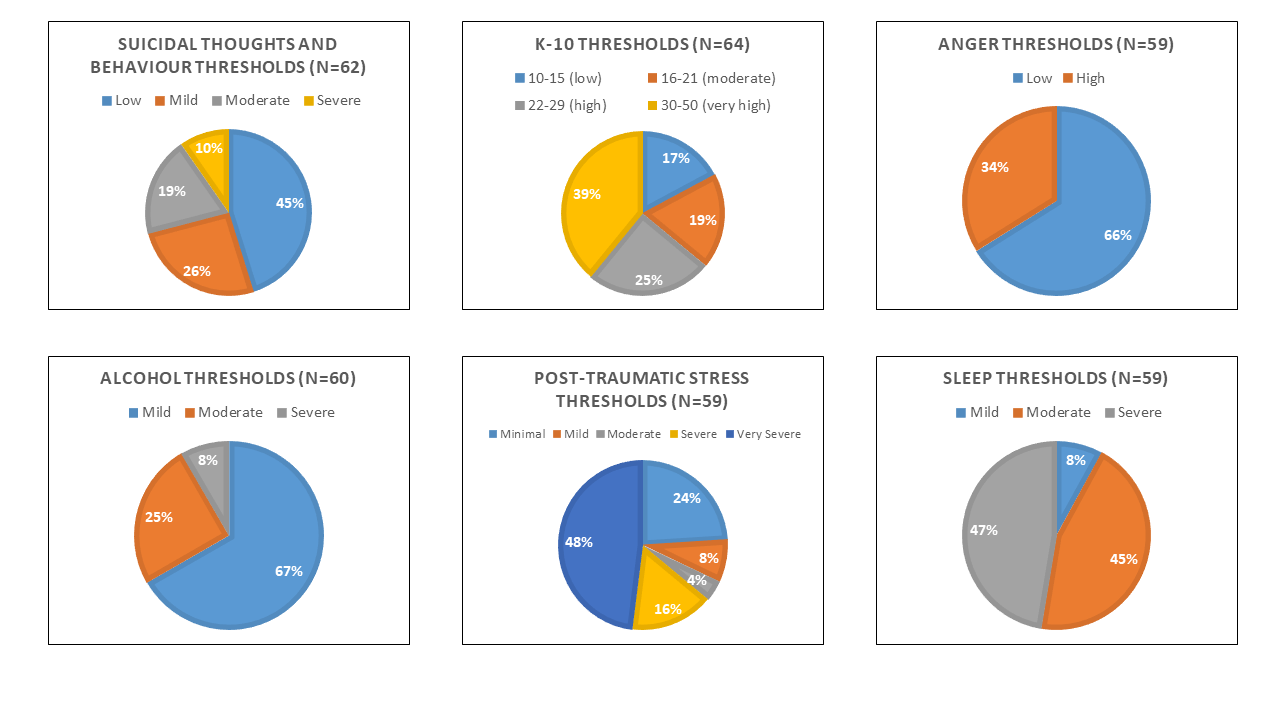


*Figure 3. Overall results from the InnoWell Platform’s multidimensional assessment at the Open Arms (Sydney and Lismore) services.*

One of the aims of the Project Synergy trial for Open Arms was to reduce suicide rates for the veteran community by using digital strategies to enhance early identification and prevention capabilities. Nearly half of the respondents (n=28/62, 45%) reported that they had no recent suicidal thoughts and behaviours; however, of those service users who did, one-quarter (n=16/62, 26%) reported mild suicidal thoughts and behaviours which would have resulted in an automatic pop-up providing contact details for 24-hour crisis services. Notably, 18 clients (n=62, 29%) reported moderate to severe levels of suicidal thoughts and behaviours, which would again have resulted in the above pop-up for the individual as well as a real-time notification to the service enabling an immediate response. Open Arms monitored the InnoWell Platform on a 24-hour basis and, as such, were able to contact all potentially at-risk service users to ensure their safety or to arrange acute care. The ability to respond immediately to the identified risk was the primary strength of the implementation of the InnoWell Platform for Open Arms.

#### NSW North Coast headspace centres (Port Macquarie, Coffs Harbour, Lismore, Tweed Heads) – Primary youth mental health service in a regional setting

The InnoWell Platform was implemented across the *headspace* services between March and June 2019, ending in December 2020. A total of 151 young people (107 females; 71%) onboarded to the InnoWell Platform. The mean age was approximately 18 years (youngest = 12 years; oldest = 25 years). Twenty (n=151, 12%) service users identified as being of Aboriginal and/or Torres Strait Islander background. Most of the young people were living with their family (n=130/151; 86%), whereas six (4%) were living on their own, seven (5%) lived in a share house, and eight (5%) reported ‘other’ living circumstances. Less than a quarter of the service users had completed at least some form of tertiary education; including a certificate or diploma (n=31/151; 21%), and/or an undergraduate degree (n=3/151; 2%). Approximately one-third (n=55/151; 35%) of young people were partially supported by government funding.

As shown in Figure 4, results from the multidimensional self-assessment indicate the majority of service users reported experiencing at least mild suicidal thoughts and behaviours (n=86/121, 71%) with approximately half of those in the high-moderate categories (n=61/121, 50%). Quite strikingly, only 11 young people reported good overall health, with approximately one-third of young people rating their overall health as poor (n=36/118, 31%). The majority of young people rated their mental health as either mildly, moderately or markedly impaired (n=86/117, 73%) with fewer rating as either good or borderline impairment (n=23/117, 20%), or severe/extremely impaired (n=8/117, 7%). Approximately one-third of the young people had impairments to everyday functioning (n=43/107, 40%), with less than one-tenth in the moderate and poor categories (n=8/107, 7%). Just over half of service users reported having moderate difficulties with social connectedness (n=63/120, 53%), with an additional one-quarter rating social connectedness as poor (n=33/120, 27%).


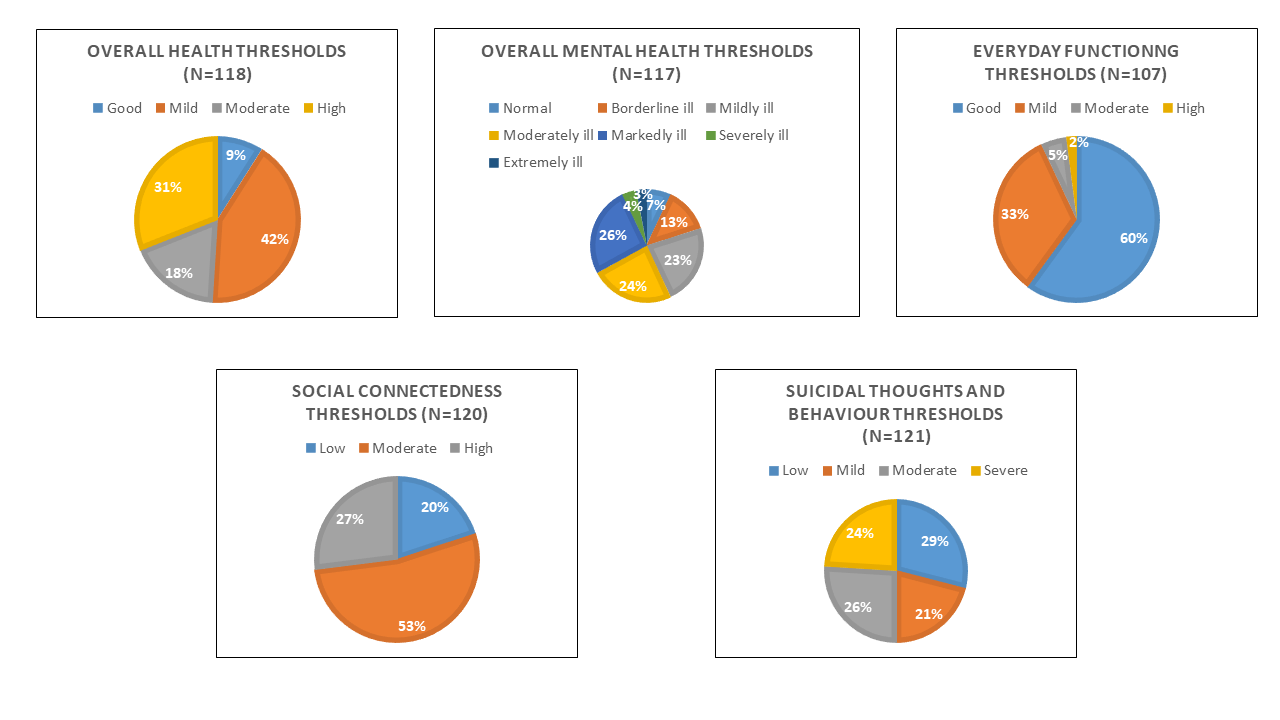


*Figure 4. Overall results from the multidimensional assessment at North Coast NSW PHN headspace services (Coffs Harbour, Lismore, Port Macquarie, Tweed Heads)*

#### The Butterfly Foundation’s National Helpline – Telephone, email, and online chat for individuals seeking support for eating disorders and negative body image

The InnoWell Platform was implemented as part of the Butterfly Foundation’s National Helpline from July 2019 until April 2020. A total of 136 service users (127 females; 92%) onboarded to the InnoWell Platform. The mean age was approximately 24 years old (youngest = 14; oldest = 66 years). Two (n=136, 2%) service users identified as being of Aboriginal and/or Torres Strait Islander background. Most service users were living with their family (n=108/136; 79%), whereas 20 (15%) were living on their own, seven (5%) lived in a share house, and one (1%) reported ‘other’ living circumstances. Almost half of the respondents (n=67/136; 48%) had completed at least some form of tertiary education; including a certificate or diploma (n=23/136; 17%), undergraduate degree (n=29/136; 20%), and/or a post-graduate degree (n=15/136; 11%). Approximately one in five (n=29/136; 20%) service users were partially supported by government funding.

As shown in Figure 5, results from the multidimensional assessment indicate that the majority of service users (n=122/127, 96%) reported experiencing body image distress and some concerning eating behaviours, which aligns with the Butterfly Foundation’s mission to support Australians impacted by eating disorders and body image issues. Physical health concerns were universal (n=128/128, 100%) as indicated by service users reporting a combination of fluctuating weight (n=107/128, 84%), low body mass indices (BMI) as defined by a BMI less than 18.5 (n=48/128, 38%), amenorrhea (n=34/128, 27%), and/or cognitive (n=126/128, 97%) and physical health symptoms (n=101/128, 79%) frequently associated with eating disordered behaviours. More than three-quarters of the contacts (n=104/136, 80%) endorsed moderate to high levels of psychological distress, with approximately half (n=71/128, 56%) reporting moderate to high levels of suicidal thoughts and behaviours. The mental health concerns experienced by service users were associated with negative effects on social and occupational functioning, with 37 (n=128, 29%) and 77 (n=128, 59%) of service users indicating a moderate or severe degree of dysfunction in education, employment or training, respectively. As described above, the mental health concerns were markedly elevated among contacts; however, more than three-quarters (n=105/128, 82%) did not report problematic use of alcohol or other substances.


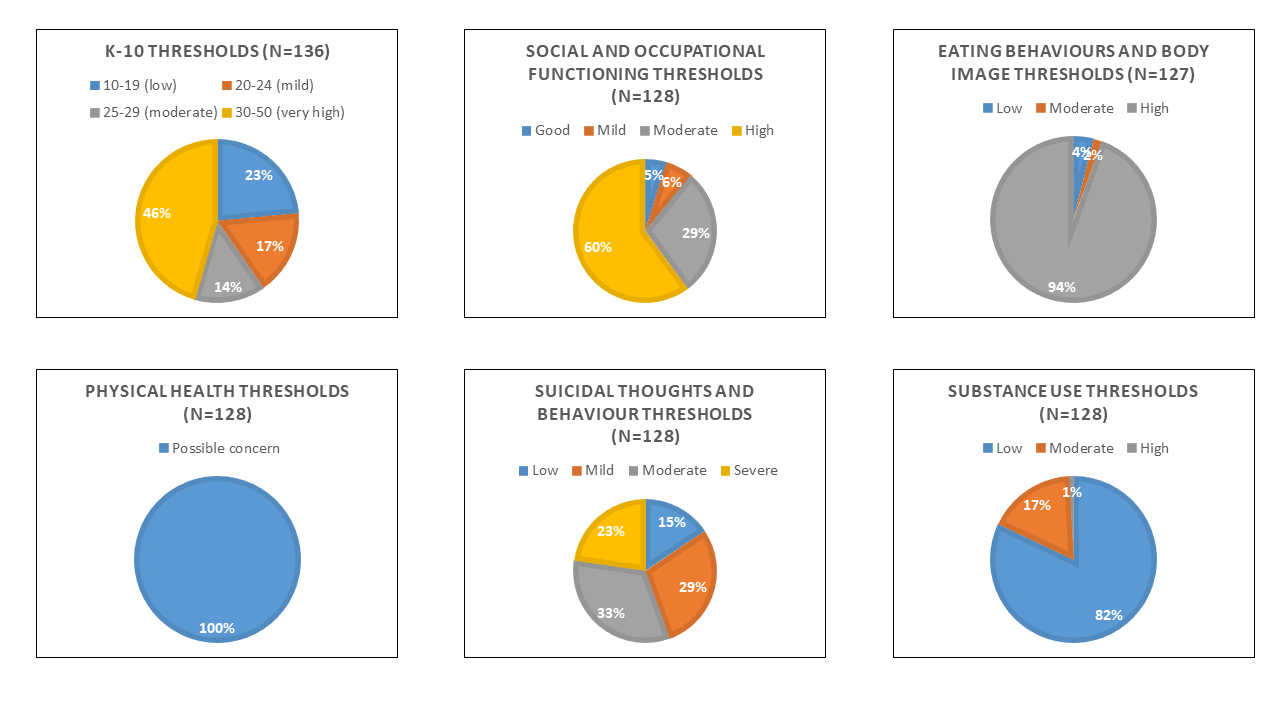


*Figure 5. Overall results from the multidimensional assessment at the Butterfly Foundation’s National Helpline.*

At the time of implementation, the InnoWell Platform was only accessible to service users via direct invitation from the Butterfly Helpline counsellors. As almost 50% of the helpline contacts are repeat callers, the service users saw the InnoWell Platform as an opportunity for contacts to avoid having *‘to keep telling their story over and over again”* (Service users with lived experience, Participatory design workshops 26 February 2019 Sunshine Coast, Queensland and 5^th^ March 2019 Darwin, Northern Territory) [38]. By storing data effectively in the InnoWell Platform, counsellors would be afforded the opportunity to better understand the service user’s needs, engage in collaborative activity planning, facilitate information provision, refer to appropriate resources, and improve overall continuity of care. In this way, the Butterfly Helpline could move towards being a technology-enabled, data-driven mental health service.

#### Connect to Wellbeing North Coast NSW (Neami National) – Community-based intake service

While not proceeding to active implementation, the partnership with Connect to Wellbeing allowed the R&D team to collaboratively co-design a prototype (see Figure 6) for a technology-enabled pre-clinic triage system. As described previously, the original prototype exhibited in Figure 6 has since been adapted to reflect its potential for national scalability at the PHN-level as shown earlier in Figure 2 [44]. A pilot implementation study is now required to evaluate the effectiveness of this model in relation to demand management broadly and mental health outcomes specifically.


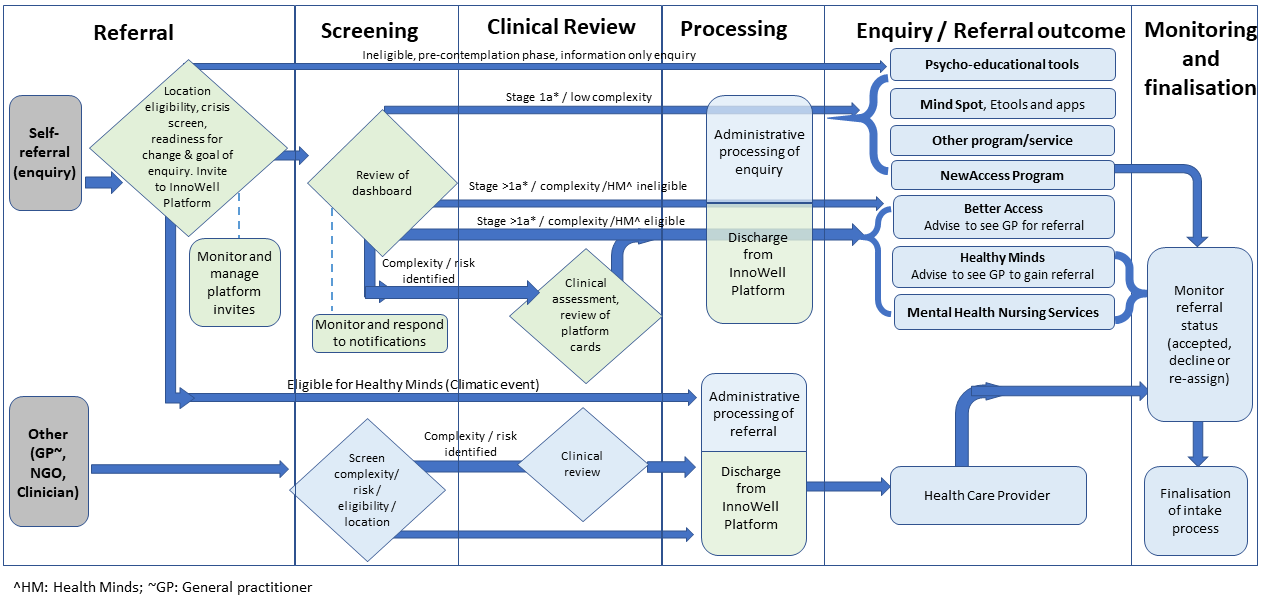


*Figure 6. Connect to Wellbeing North Coast – Technology enhanced process pathway for implementation.*

#### Kildare Road Medical Centre – Large-scale general practice

As family practices are often the first port of call for Australians seeking mental health care, we established a strategic partnership with KRMC. The InnoWell Platform was implemented from December 2019 until December 2020. A total of 136 service users (77 females; 57%) completed the onboarding process. The mean age was approximately 35 years (youngest = 14; oldest = 72 years). Fourteen (n=136, 9%) service users identified as being of Aboriginal and/or Torres Strait Islander background. Most service users were living with their family (n=107/136; 79%), whereas 19 (14%) were living on their own, three (2%) lived in a share house, one (1%) reported being homeless, and six (4%) reported ‘other’ living circumstances. Less than half of the respondents had completed any form of tertiary education; certificate or diploma (n=32/136; 23%), post-graduate degree (n=11/136; 8%), and undergraduate degree (n=17/136; 12%). Approximately one-quarter (n=37/136; 27%) of service users were partially supported by government funding, with an additional 20 (14%) being fully dependent on government funding.

As shown in Figure 7, results from the multidimensional assessment indicate that one-third (n=61/128, 35%) of service users report being in good overall health; however, two-thirds of service users (n=91/136, 67%) endorsed moderately severe general health concerns. Overall mental health was highly variable, ranging from *‘normal’* (n=28/135, 20%) to *‘moderately ill’* (n=31/135, 23%) to *‘extremely ill’* (n=3/135, 2%). Strikingly, nearly half (n=62/136, 46%) of service users reported very high levels of psychological distress. Furthermore, 17 (n=74, 23%) of service users endorsed high levels of suicidal thoughts and behaviours, resulting in an immediate escalation to the service, and an additional 57 (n=74, 77%) reported mild to moderate levels of suicidal thoughts and behaviours which would have triggered a pop-notification with the contact details for 24-hour crisis support services. Although half of the service users noted that they consumed low levels of alcohol, 14 (n=90, 15.6%) service users endorsed problematic alcohol use, leaving them vulnerable to the social, health, financial and/or legal consequences of alcohol use.


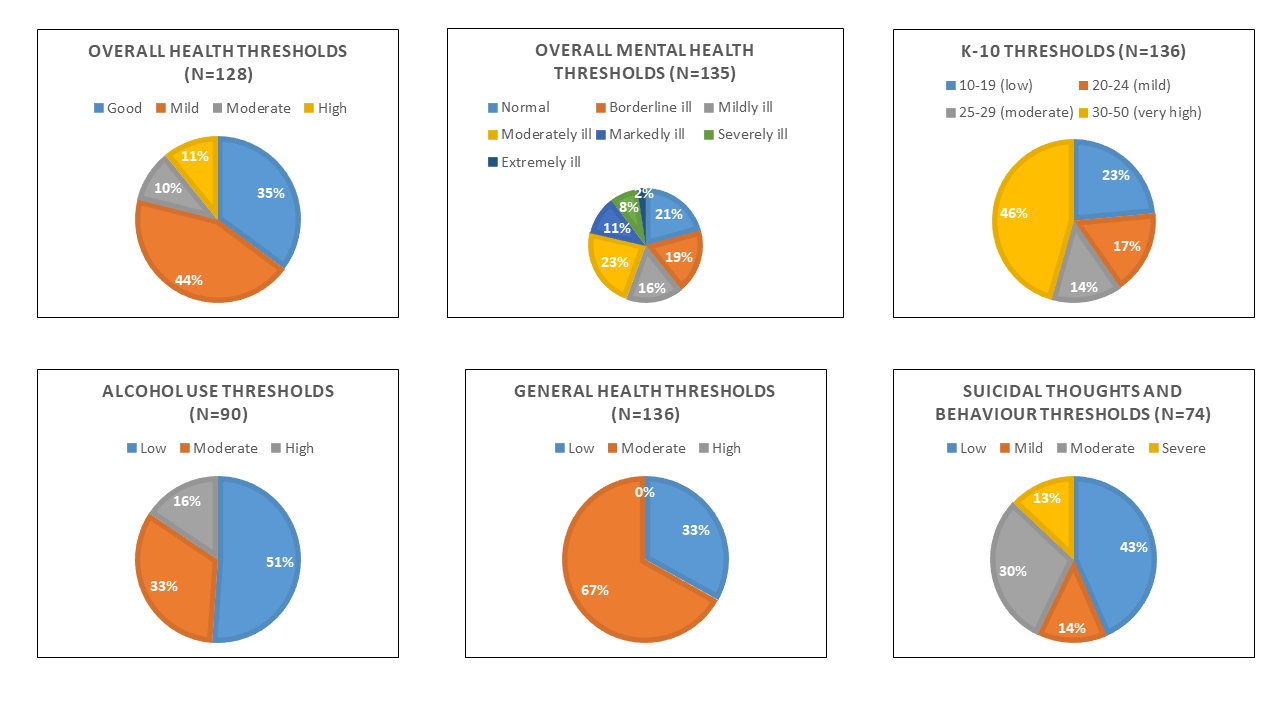


*Figure 7. Overall results from the multidimensional assessment at Kildare Road Medical Centre.*

Importantly, as shown in Figure 8, KRMC’s configuration of the InnoWell Platform took advantage of the ability to tailor the question sets based on a service user’s age (i.e., 14-49 years old and 50 years and older). For example, questions related to functioning for younger service users focused on participation in education and employment. Aggregate data indicates that approximately two-thirds (n=68/101, 66%) of younger service users are experiencing moderate to high levels of impairment in social and occupational functioning. Older adults were asked about their ability to complete instrumental activities of daily living, and again, approximately two-thirds (n=19/28, 69%) reported moderate to high levels of difficulty in this health domain. Further development is now required to enable greater personalisation of the InnoWell Platform’s multidimensional assessment based on demographic variables as well as in relation to clinical presentation.


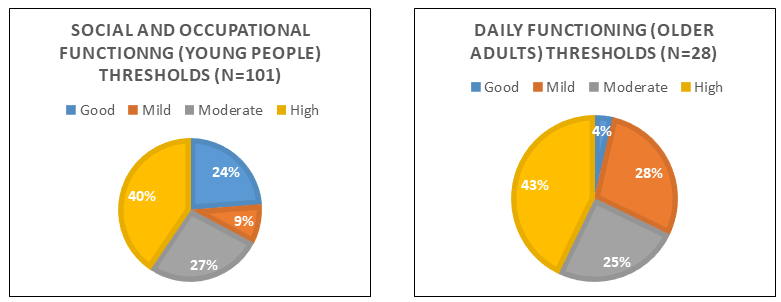


*Figure 8. Aggregate functional profiles of KRMC service users based on age.*

#### Older Adults

Four participatory design workshops were conducted to explore how adults aged 50 years and older are using technology in general, and to identify the potential barriers to and facilitators of the adoption of digital solutions by this cohort to support health and wellbeing. As previously reported, our findings indicate that technology use is widespread among older adults [39]. Additionally, the participants consistently indicated that they were more likely to use digital solutions that includes personalised content, functionality that responds to the service user’s needs, and up to date information from credible sources. Finally, it was noted that digital solutions need to integrate well as part of standard care practices to facilitate the therapeutic relationship with health professionals [39].

The findings from the above study were subsequently used to configure a prototype of the InnoWell Platform for older adults, including modifications to health domains, informational material, and care options. To evaluate the usability, acceptability and engagement of this prototype, community dwelling older adults, independent of a specific mental health care service and recruited through the University of Sydney’s Brain and Mind Centre, engaged with the InnoWell Platform naturalistically (i.e. at their own discretion) for a period of 90-days. After 90 days, participants were asked to complete the System Usability Scale to evaluate the usability and acceptability of the prototyped InnoWell Platform. Overall, participants found the InnoWell Platform easy to use and valued the comprehensive nature of the assessment tools; however, further evaluation with help seeking older adults is required [40].

#### Children and Families

Three participatory design workshops were conducted to explore the potential uses of HITs with children and their families to support mental health and wellbeing. A total of 15 participants recruited through the University of Sydney’s Brain and Mind Centre consented to participate in the workshops, including six parents/guardians, two support persons and seven children (ranging from 8 to 13 years of age). The workshops varied in duration, running up to 3 hours in length, and method of delivery (i.e. face-to-face vs Zoom).

The findings from the workshops highlighted that technology use is prevalent among this younger group, with a preference for iPads/tablets and smartphones. Participants were accessing a range of apps and e-tools, including those for meditation, mental health (e.g., worry box), fitness tracking, yoga, nutrition and blood sugar monitoring, social media and messaging, as well as telephones for communication, learning tools (i.e. handwriting, reading eggs), and digital platforms to message professionals. The COVID-19 pandemic and associated lockdowns resulted in an increase in technology use, including for the purposes of digital health; however, it was noted that it was difficult to find privacy in home. Interestingly, children indicated that they preferred face-to-face appointments with counsellors, whereas digital health was preferred for more general medical appointments, such as with a family doctor.

In relation to privacy and data sharing, children were happy for parents to have shared control over their personal and health information until they were 15 or 16 years old. Furthermore, they welcomed parental input from 9 to 10 years old, but indicated that parents/guardians should have full control of the data and information for children aged 8 years and younger. Importantly, some schools provided training regarding safe online behaviour. Parents understood that they were not able to completely monitor everything their child does online and were aware that their children may hide their engagement with some content. To minimise these risks, some parents were using various online tools or authentication and security on google play store, to control and monitor what is downloaded and viewed. It was noted that proper education regarding privacy, technology and mental health is critical for both parents/guardians and children. It was recommended that services have clinical guidelines in place to help parents make decisions about what is appropriate for their child. Information sharing (e.g., data sharing with a teacher) was also deemed to be very important; however, participants noted that there should always be a choice as to what details are shared and what personal and health information remains private.

Textbox 2 highlights the features that the children wanted to be included in a digital tool they might use to support their health and wellbeing.

*Textbox 2. The features that children highlighted as being important for HITs*

| Features to include in HITs:   - - - - Private calls with health professionals       - Emergency alerts/notifications       - The option to choose three trusted contacts       - Achievement scores (reward/motivation system)       - Music to help you calm down       - Mindfulness videos       - Activity tracker, tracking over time       - Health checks and advice on health. |
| --- |

When asked to provide feedback on the InnoWell Platform, the children noted that they liked the colours, found it easy to understand, and thought it was well laid out (i.e., not too crowded). They agreed that tracking personal data over time is important to be able to see your mental health journey. They indicated that they were likely to update health cards fortnightly or monthly, depending on mood changes. Finally, they were happy to invite support people, including parents and teachers, to provide an informant’s perspective via the InnoWell Platform. Parents also provided specific feedback on the InnoWell Platform, indicating that it should focus on a child’s strengths as well as areas in which they can improve. They also noted that it needs to look fun, use age-appropriate language (i.e. not be overly clinical), and include images and colours so it is easily understood.

### References

Weathers FW, Litz BT, Keane TM, Palmieri PA, Marx BP, Schnurr PP. The PTSD Checklist for DSM-5 (PCL-5). 2013. Scale available from the National Center for PTSD at [www.ptsd.va.gov](http://www.ptsd.va.gov).
